# Effects of Biodegradation of Corn-Starch–Sodium-Alginate-Based Liquid Mulch Film on Soil Microbial Functions

**DOI:** 10.3390/ijerph19148631

**Published:** 2022-07-15

**Authors:** Xia Gao, Chenxing Fu, Mingxiao Li, Xuejiao Qi, Xuan Jia

**Affiliations:** 1 State Environmental Protection Key Laboratory of Food Chain Pollution Control, Beijing Technology and Business University, Beijing 100048, China; 18793906968@163.com (X.G.); fuchenxing718@163.com (C.F.); 2 Key Laboratory of Cleaner Production, Integrated Resource Utilization of China National Light Industry, Beijing Technology and Business University, Beijing 100048, China; 3 State Key Laboratory of Environmental Criteria and Risk Assessment, Chinese Research Academy of Environmental Sciences, Beijing 100012, China; limingxiao8122@163.com (M.L.); qixj19@mails.tsinghua.edu.cn (X.Q.)

**Keywords:** liquid mulch, corn starch, sodium alginate, soil microbial, biodegradation

## Abstract

In response to the problems of the poor degradability and mechanical properties of liquid mulch, natural non-toxic polymer compound corn starch and sodium alginate were used to prepare fully biodegradable liquid mulch. The preparation conditions of the mulch were optimized, and the mechanical properties of the mulch and the changes in the microbial community in soil with the mulch degradation were analyzed. The corn-starch–sodium-alginate-based liquid mulch film had an optimum performance at a tensile strength of 0.145 MPa and an elongation at a break of 16.05%, which was attained by adding 33.33% sodium alginate, 50% glycerol 22 and 4% citric acid to corn starch after moist heat modification. Fourier transform infrared spectroscopy analysis showed that the -COOH in sodium alginate could interact with the -OH in starch and glycerol through hydrogen bonding, thus, resulting in a denser structure and better mechanical properties of the liquid mulch as a non-crystalline material. The soil burial degradation study of mulch revealed that corn-starch–sodium-alginate-based liquid mulch degraded completely at 25 days macroscopically, and mulch degradation increased soil organic matter content. Microbial kinetic analysis showed that the abundance and diversity of the bacterial community decreased with the degradation of the mulch, which was conducive to the optimization of the bacterial community structure and function. *Arthrobacter* of the class *Actinomycetes* became the dominant microorganism, and its abundance increased by 16.48-times at 14 days of mulch degradation compared with that before degradation, and *Acidophilus* phylum (14 days) decreased by 99.33%. The abundance of fungal communities was elevated in relation to the main functional microorganisms involved in liquid mulch degradation, with *Alternaria* and *Cladosporium* of the *Ascomycete* phylum *Zygomycetes* being the most active at the early stage of mulch degradation (7 days), and the relative abundance of *Blastocystis* was significantly elevated at the late stage of mulch degradation (14 days), which increased by 13.32%. This study provides important support for the green and sustainable development of modern agriculture.

## 1. Introduction

Mulch films have been used for ground covering to retain heat and water, improve soil moisture, maintain soil structure, and prevent weeds and pests. However, the “white pollution” caused by the widespread application of mulch films is serious. The development of new environmentally friendly biodegradable mulching is of great significance to mitigating the greenhouse effect and surface pollution and ensuring food safety.

Biodegradable mulch is difficult to fully degrade [1] and needs careful laying in the field as it sometimes gets broken during soil application. As a new biodegradable soil cover material, the liquid mulch film could be more conducive to soil than traditional plastic films. The liquid film can form a film on the surface layer of soil, wrap the soil pores, limit the evaporation of soil moisture, play the role of heat preservation, and cement the dispersed soil particles. What is more, it can be sprayed on soil, which could save abundant efforts in agriculture practice. After the agriculture harvesting, the used mulches can be biodegraded in soil that will not need any treatments, which has become a research hotspot [2,3,4,5,6].

Recent research has studied materials used for the preparation of biodegradable liquid mulch, including chemical polymers, natural polymers, and composite materials. Natural polymers have excellent degradability, safety, permeability [2,7,8], and economy among these materials. However, the film-forming properties have problems, such as poor mechanical properties and rapid degradation rate. Majeed et al. [8]. mentioned the application of biodegradable polymers and their blends in controlled-release fertilizers, including some natural polymers, especially lignin, starch, chitosan, fucoidan, cellulose, or their modified forms of various polymers, which were important means to reduce costs, improve marketability, and protect land fertility. Starch, as a natural polymer with a wide range of applications and sources, was easily processed and biodegradable. Sodium alginate was an abundant hydrophilic natural polymer with good film-forming and adsorption properties. The application properties of oxidized-corn-starch–gelatin (OCS-Gel), as a liquid mulch for moisture absorption, permeability, and water retention, were explored. Dang et al. [9]. found that the OCS-Gel enriched the pore structure and the presence of cross-linkage in the composite made it highly compatible, thermally stable up to 324.8 °C, and the germination rate reached 80%, exhibited good water absorption, water retention properties, and low permeability. Adhikari et al. [10]. stated that when studying the conditions under which sprayable biodegradable polymer liquid mulch can maximize agricultural water use efficiency, it was crucial to know the fundamental relationships and interactions between polymer chemistry, structure and properties, and soil chemistry, physics, and biology. The microbial community structure was crucial for regulating and maintaining soil ecosystem function and was a sensitive indicator for assessing the function of contaminated soil ecosystems [11,12]. Sodium-alginate-based solutions were used as liquid mulch films and the effects of the mulch on the soil properties were studied [13]. Sen et al. [14]. studied the biodegradability of starch-based self-supporting antimicrobial membranes and their effects on soil quality with the soil burial method and found that biodegradation reached 90% within 28 days; biodegradation kept the soil pH within the normal tolerance range for plant growth and increased the soil organic carbon, total nitrogen, effective nitrogen, and water-holding capacity. Hence, the biodegradation of the membranes improved soil quality and influenced microbial communities. To explore the bacterial community succession within the plastic layer of polyethylene mulch, Wang et al. [15]. found that the community composition in the plastic mulch layer and the surrounding liquid differed from that of the original soil, in which *Amoeba* and *Bacteroides* were enriched at the phylum level and *Pseudomonas* and *Methylotrophomonas* were enriched at the genus level. It has been proved that the use of mulch affected the structure and function of soil microbial communities. However, little research has been conducted on the effects of liquid mulch usage and degradation processes on soil microbial communities and their degradation mechanisms.

The aim of this study was to develop a new fully biodegradable liquid mulch and to find the response mechanisms between liquid mulch degradation and soil microorganisms. Natural polymers (corn starch and sodium alginate), as the main raw materials, were used to prepare biodegradable liquid mulch. The physicochemical properties of corn-starch–sodium-alginate-based liquid mulch were analyzed. Functional groups and crystal structure were also analyzed by Fourier transform infrared spectroscopy (FTIR) and X-ray diffraction (XRD). Soil burial degradation experiments were performed to examine and investigate degradation properties and the relationship between the mulch degradation process and the response of soil microbial succession. This study will provide a theoretical basis and technical support for the application and promotion of degradable liquid mulch.

## 2. Materials and Methods

### 2.1. Materials

The reagent-grade corn starch (C_6_H_10_O_5_)n and glycerol (C_3_H_8_O_3_) were purchased from Shanghai Maclean Biochemical Technology Co., Ltd. (Shanghai, China). Chemically pure sodium alginate (C_6_H_7_NaO_6_)n was purchased from Sinopharm Chemical Reagent Co., Ltd. (Beijing, China). Analytical pure citric acid (C_6_H_8_O_7_) was obtained from the Tianjin Jinke Fine Chemical Research Institute (Tianjin, China), and industrial grade Vaseline(C_6_H_14_O_2_) was from Fuchen (Tianjin) Chemical Reagent Co., Ltd. (Tianjin, China).

Soil was collected from the topsoil of a greenhouse in Shouguang City, Shandong Province, China. The soil properties in the top 20 cm were as follows: soil organic matter at 40.85 g/kg, pH at 8.1, electrical conductivity at 0.34 ds/m, and total dissolved salt at 6.68 g/kg.

### 2.2. Experimental Design

#### 2.2.1. Preparation of Liquid Mulch Film

The modification of natural starch using hydrothermal treatment is an environmentally friendly physical modification method. It can rearrange the internal molecules of the starch granules [16] and partially pastes the starch granule. In addition, it will enhance the intermolecular interactions between starch molecules, improving the water resistance of starch and its resistance to enzymes.

**Figure 1:**
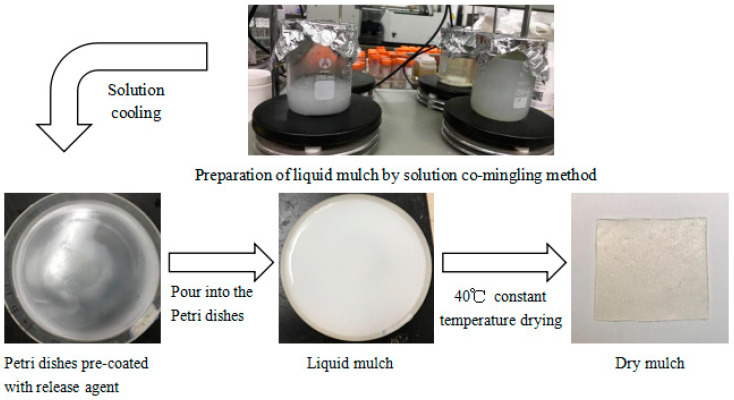
Modified corn starch and sodium alginate base film preparation process.

It was concluded that a treatment humidity of 25%, a treatment temperature of 120 °C, and a treatment time of 8 h were the optimal hygrothermal frontal reaction conditions. The appropriate amount of hydrothermally modified corn starch was weighed in a beaker and stirred until the starch was completely pasted [17], dissolved sodium alginate [18] was added, and then citric acid and glycerol were added and stirred at 400 rpm/min for 2 h to obtain the liquid ground film. The liquid mulch film was formed by casting and the preparation process is shown in Figure 1.

The optimization of conditions for liquid mulch preparation was carried out by single-factor experiments, based on the pre-experiment setting of 3 factors, each with 5 levels, and the reaction parameters were set as shown in Table 1.

#### 2.2.2. Liquid Mulch Film Degradation and Soil Burial Experiment

A degradability test was carried out after the liquid mulch film was formed by casting and was air-dried [19]. It was buried at a depth of 10 cm in the soil (pH 8.1, soil moisture 30%, temperature 25 °C). The mulch was removed and dried on days 0, 7, 14, and 21 to determine the weight loss rate of the mulch, and soil samples were collected using the five-point sampling method to determine the soil organic matter content and microbial community structure. Samples for microbial community structure determination were stored at −80 °C for backup and determined by high-throughput sequencing. Five-point sampling method was adopted for soil sampling. First determine the midpoint of the diagonal as the central sampling point and then select four points on the diagonal with the same distance from the central sampling point as the sampling point.

### 2.3. Analysis Methods

The mechanical properties of the mulch were determined using a microcomputer-controlled electronic universal testing machine (CMT4304, test speed 10 mm/min, sensor 50 N, Putian, China) [20,21].

The functional groups of the ground membrane were analyzed using a Fourier transform infrared spectrometer (Tensor II, Bruker, Germany) using KBr pellets at a mass ratio of 1:100 [22]. The geomembrane crystal structure was analyzed by an X-ray diffractometer produced by Rigaku corporation (Ultima IV, Tokyo, Japan) with a tube pressure of 40 kV, a tube current of 100 mA, a scan speed of 2°/min, and a scan range of 2θ using Cu Ka radiation at a 2θ range from 5° to 70° with a scan step size of 0.02°. X-ray energy spectrum (EDS) was used in conjunction with scanning electron microscope and transmission electron microscope to analyze the types and contents of elements in the micro area of materials.

The microbial analysis of soil community composition was determined by high-throughput sequencing. The bulk soils were collected from the place where the dry mulch film was buried and also kept in sterile plastic bags. The soil samples were immediately transported in an ice-packed container to the laboratory and stored in a cold room at 4 °C prior to analysis. Microbial community genomic DNA was extracted from soil samples and bacteria were amplified by forwarding primer 338F (5′-ACTCCTACGGGAGGCAGCAG-3′) and reverse 806R (5′-GGACTACHVGGGTWTCTAAT-3′) for the V3-V4 region of the 16S rRNA gene. The amplification of the fungus was performed in the ITS1F_ITS2R region by PCR reactions with the forward primer ITS1F (5′-CTTGGTCATTTAGAGGAAGTAA-3′) and the reverse primer ITS2R (5′-GCTGCGTTCTTCATCGATGC-3′). PCR reactions were repeated three times, and PCR products were extracted from 2% agarose gels, purified with the AxyPrep DNA Gel Extraction Kit (Axygen Biosciences, Axygen, Union City, CA, USA), and quantified using a Quantus^TM^ fluorometer (Promega, Madison, WI, USA). Alpha diversity index, colony structure, and function prediction analysis were performed on Meguiar’s Bioinformatics Cloud platform (https://login.majorbio.com, accessed on 2 April 2021). Simpson indices were used to calculate species richness and relative abundance (alpha diversity), while Shannon indices were used to calculate species similarity (beta diversity) in different sampling units. Both of them can describe species richness and diversity. Chao indices were used to estimate the number of OTUs contained in a sample. The higher its value, the more species in the sample. Ace indices were used to evaluate the richness and evenness of species composition in the sample. The larger its value, the richer the species in the environment and the more uniform the distribution of species.

### 2.4. Calculation Method

The weight loss rate of the mulch was calculated according to Equation (1), and the corn-starch–sodium-alginate-based dry mulch was prepared as shown in Figure 1, where WL is the weight loss rate of the mulch, M_0_ is the dry weight of the mulch before degradation (dried to constant weight at a constant temperature of 40 °C), and M_1_ is the dry weight of the mulch after degradation [23].

WL = (M_0_ − M_1_)/M_0_ × 100% (1)

## 3. Results and Discussion

### 3.1. Performance of Corn-Starch–Sodium-Alginate-Based Liquid Mulch

#### 3.1.1. EDS Analysis of Corn-Starch–Sodium-Alginate-Based Liquid Mulch

Table 2 shows the main elements in the liquid mulch, as well as the mass ratio and atomic ratio of each main element. It can be seen that the liquid film is mainly composed of C and O elements, which are derived from each raw material for the synthesis of the liquid film. Among them, the content of element C is the highest, accounting for 58.09% of the total mass, and the number of atoms accounts for 64.94% of the whole, indicating that the liquid mulch is a carbon skeleton organic polymer. Element O is also an element constituting the liquid mulch film, and its mass and number of atoms account for 41.43% and 34.78% of the total, respectively. Na comes from sodium alginate with less content.

#### 3.1.2. Tensile Strength and Elongation at Break Properties

**Figure 2:**
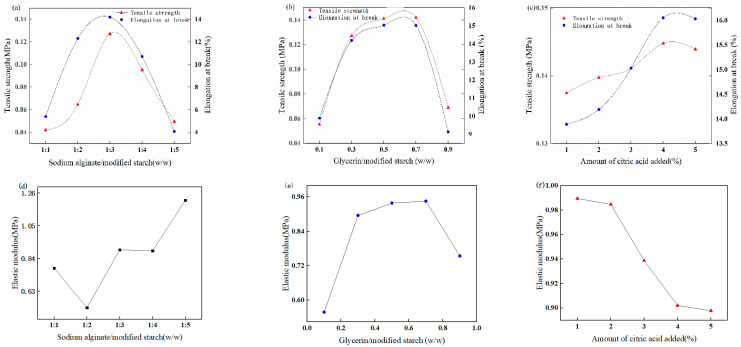
Effect of sodium alginate addition on the mechanical properties of the dry film (**a**), effect of glycerol addition on dry film mechanical properties (**b**), effect of citric acid addition on the mechanical properties of the dry film (**c**). Effect of sodium alginate addition on the elastic modulus of the dry film (**d**), effect of glycerol addition on dry film elastic modulus (**e**), effect of citric acid addition on the elastic modulus of the dry film (**f**).

In this study, the tensile strength and elongation at break of air-dried mulch were used as evaluation indices to optimize the conditions for the preparation process of liquid mulch, as the mechanical properties of liquid mulch are the critical constraints for its application. The effects of different additions of sodium alginate, glycerol, and citric acid on the tensile strength and elongation at the break of the mulch film are shown in Figure 2.

As shown in Figure 2a, the elongation at break and tensile strength of the mulch film exhibited a trend of increasing and then decreasing with the addition of sodium alginate. When the ratio of sodium alginate to starch was 1:3, the tensile strength reached a maximum value of 0.13 MPa, the elongation at break reached a maximum value of 14.19%, and the elongation at break and tensile strength decreased when changing the ratio of both. The improvement in mechanical properties is due to more hydrogen bonds formed between sodium alginate and starch as the content of sodium alginate increases, so the network structure of the polymer is more stable. The weaker mechanical properties may be attributed to the excess of sodium alginate and the decrease in starch content, as only a small amount of starch can combine with sodium alginate, Hence, the hydrogen bonding content between the two is reduced and the mechanical properties are lower.

As illustrated in Figure 2b, the elongation at break and tensile strength of the mulch film showed a trend of increasing and then decreasing with the addition of glycerol. The tensile strength reached a maximum value of 0.14 MPa and the elongation at break reached a maximum value of 15.02% when the mass ratio of sodium alginate to starch was 1:3 and the mass ratio of glycerol to modified starch was 0.7, similar to the test results when the mass ratio was 0.5. Therefore, the mass ratio of glycerol to starch was chosen to be 0.5 to maintain the optimized conditions, considering the production cost. Glycerol is a commonly used plasticizer that can improve the flexibility of the mulch material by forming hydrogen bonds with starch molecules through hydroxyl groups, thus, changing the molecular structure of the starch [24]. 

As presented in Figure 2c, the tensile strength reached a maximum value of 0.16 MPa, and the elongation at break reached a maximum value of 16.05% when the mass ratios of sodium alginate to starch and glycerol to modified starch were 1:3 and 0.7, respectively, and the addition of citric acid was 4%. It showed that citric acid played a certain cross-linking role during the reaction process, which made the macromolecules combine effectively and improved the mechanical properties of the mulch film. The elongation at break and tensile strength of the mulch film was reduced when the amount of citric acid added was greater or less than 4%, owing to the combination of excess citric acid and starch, which led to increased difficulty in the intermolecular slip of starch, resulting in lower elongation at break and tensile strength [25]. Citric acid is a tri-carboxylic acid used to cross-link polysaccharide molecules with each other. In addition, citric acid enhances the intermolecular interactions of liquid mulch and improves the performance of the mulch. However, the high content in citric acid and the consequent acid hydrolysis depolymerize the starch molecules into smaller polysaccharides and promote the destruction of the starch granules [26].

It was observed that the best mechanical properties of the liquid ground film were prepared by choosing 1:3 sodium alginate/modified starch (*w*/*w*), 1:2 glycerol/modified starch (*w*/*w*), and 4% citric acid addition.

#### 3.1.3. Functional Group Analysis by FTIR

**Figure 3:**
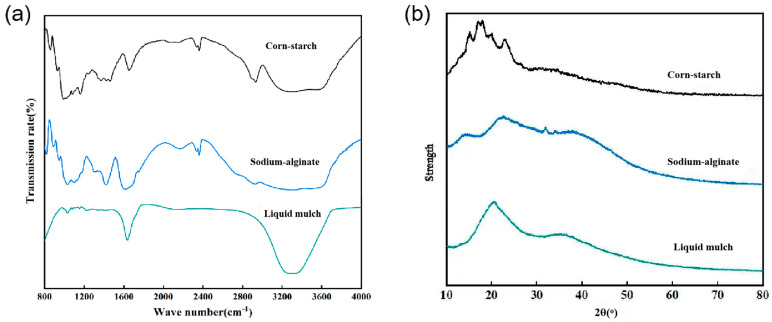
FTIR spectra (**a**) and XRD diffraction images (**b**) of liquid mulch and its main raw materials.

The functional group analysis was conducted on the solid starch and alginate films. The distribution of functional groups of liquid mulch and its components are shown in Figure 3a, which shows that liquid mulch is significantly different from modified starch and sodium alginate. Compared to modified starch and sodium alginate, the modified corn-starch–sodium-alginate-based liquid mulch has a stronger peak intensity at 3600–3000 cm^−1^ with reduced fine structure due to -OH stretching vibrational peaks in the corn starch and glycerol structures [27]. The presence of ester groups at 1720 cm^−1^ in the liquid mulch is created by combining citric acid with other polymers, indicating that crosslinks are formed. The carboxyl (-COOH) stretching vibration peaks of alginate appearing at 1640–1400 cm^−1^ (Wilpiszewska et al., 2019) and the absorption peaks between 1200 and 1000 cm^−1^ caused by the C-O stretching vibration of the glycosidic bond [28] are generated by the addition of sodium alginate. The -COOH in sodium alginate can interact with the -OH in starch and glycerol through hydrogen bonding to make the liquid ground film more compact and, thus, better serve to inhibit water evaporation and increase the effective temperature accumulation.

#### 3.1.4. Geomembrane Crystal Structure Analysis by XRD

The XRD spectra for the two main raw materials of corn-starch–sodium-alginate-based liquid mulch and prepared mulch were obtained for the solid starch and alginate films and are shown in Figure 3b. The characteristic crystalline diffraction peaks of corn starch after moist heat treatment were weakened and the crystallinity was reduced, but the crystalline structure remained. Sodium alginate showed two weak crystalline diffraction peaks at 32.90° and 34.06°, which proved that a small amount of crystalline structure was also present in sodium alginate. The corn-starch–sodium-alginate-based liquid mulch only showed a broad and strong non-crystalline peak near 20.68°, indicating that the crystal structures of modified corn starch and sodium alginate were gradually destroyed during the preparation of the liquid mulch and the mulch had obvious non-crystalline structures. Research has shown that the varied microstructure of the non-crystalline materials can be assigned to the irregular arrangement of particles. The non-crystalline structure characteristics gradually appear when the crystal structure is reduced, thus, enhancing the transparency, impact strength, and ductility of the mulch [29].

### 3.2. Degradation Performance of Corn-Starch–Sodium-Alginate-Based Liquid Mulch

**Figure 4:**
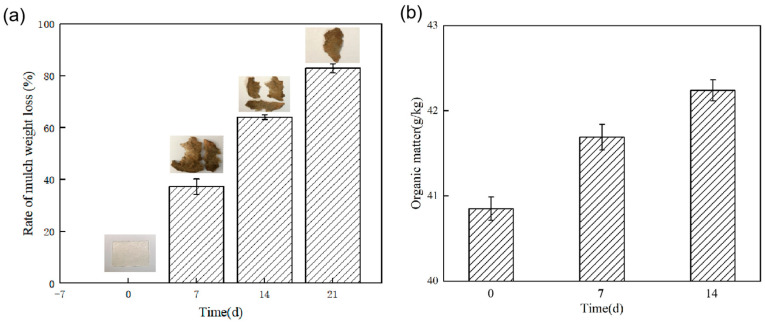
The rate of mulch weight loss under landfill experiment (**a**), changes in soil organic matter over time after application of liquid mulch (**b**).

The degradation rate of corn-starch–sodium-alginate-based liquid mulch film over time is shown in Figure 4a. The results showed that the mulch was gradually degraded with the extension of soil burial time. The mulch film weight was 20.12 g at 0 days, 12.63 g at 7 days, 7.21 g at 14 days, and 3.43 g at 21 days, the degradation rates were 37.23%, 64.17%, and 82.95% at 7 days, 14 days, and 21 days, respectively. Under the joint action of soil microorganisms and moisture, the mulch morphology changed from a large volume of dry mulch into small fragments; dry corn-starch–sodium-alginate-based mulch completely disappeared macroscopically on day 25. 

As presented in Figure 4b, the soil organic matter of the control group without liquid mulch application was 40.85 g/kg, and the organic matter content increased by 2.06% and 3.40% at 7 days and 14 days of mulch application, respectively, compared to the control group. Pronk et al. [30] analyzed the relationship between the role of soil organic matter and microorganisms and showed that microorganisms are responsive to changes in organic matter. As an essential nutrient in the growth of microorganisms, small-molecule carbon in organic matter can effectively promote microbial reproduction and metabolism in soil.

### 3.3. Response of Liquid Mulch Degradation to Soil Microbial Community Structure

#### 3.3.1. Abundance and Diversity of Soil Microbial Communities

The Shannon and Simpson’s indices showed in Table 3. The results indicated an overall decreasing trend with the degradation of liquid mulch, while the Ace and Chao indices showed different trends for bacteria and fungi.

The Shannon and Simpson indices indicated a gradual decrease in bacterial community diversity, with a 52.85% decrease in bacterial community diversity. The Ace and Chao indices demonstrated that the bacterial community richness in soil decreased with the continuous degradation of liquid mulch and the bacterial community richness decreased by 48.89% after 7 days of degradation.

The Shannon and Simpson’s indices in fungal community diversity had no significant change after 7 days of liquid mulch application compared to before. The Ace and Chao indices in fungal community richness showed an increasing trend. The fungal richness increased by 31.33% compared with before at 14 days of degradation. 

There were 301,924 valid sequences after filtering and the coverage rate of all samples was higher than 99% in this study, which showed that the mulch degradation was conducive to the growth and reproduction of fungi and optimization of bacterial functions.

#### 3.3.2. Community Succession of Soil Bacteria

**Figure 5:**
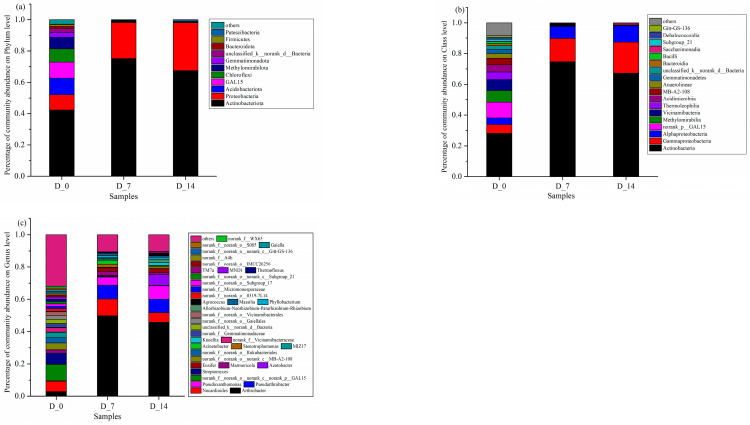
Community composition of bacteria at phylum level (**a**), class level (**b**), and genus level (**c**).

The soil samples at 0, 7, and 14 days of mulch degradation were labeled as D_0, D_7, and D_14, respectively. As shown in Figure 5a, the dominant phyla observed in all treatment groups were *Actinobacteria*, *Proteobacteria*, and *Acidobacteria*. The relative abundance of *Actinobacteria* and *Proteobacteria* increased from 42.24% in the control group to 75.18% and 67.54% at days 7 and 14 of the mulch degradation, respectively. As suggested by a previous study [31], the heat treatment of maize starch with citric acid as a cross-linking agent resulted in resistant dextrins that promoted the growth of *Actinobacteria*, which are producers of compounds that are essential for human and animal health by decomposing soil organic matter, contributing to the global carbon cycle and increasing plant productivity [32]. The phylum *Proteobacteria* increased from 9.92% in the control to 23.05% and 30.68% at days 7 and 14, respectively. The increase in the abundance of the phylum *Metamorphomonas* also indicates an improvement in soil quality (Araujo et al., 2020). It colonizes high-nutrient environments rich in carbon and is complex trophic. The relative abundance of *Acidobacteria* phylum decreased from 10.50% in the control to 0.28% and 0.07% at 7 days and 14 days of mulch degradation, respectively. *Acidobacteria* is suitable for survival in acidic environments and low-nutrient soil conditions and can be used as an indicator of soil impoverishment [33]. Furthermore, *Actinobacteria* and *Proteobacteria* gradually replaced *Acidobacteria* as the dominant flora in the soil after mulching, indicating that the soil quality improved after mulching.

As presented in Figure 5b, the dominant soil bacterial communities were *Actinobacteria*, *Alphaproteobacteria*, and *Gammaproteobacteria* of the phylum *Aspergillus* at the phylum level. The most significant changes in *Actinobacteria* were observed at 7 days and 14 days after the application of liquid mulch, with the relative abundance of *Actinobacteria* increasing from 28.22% in the control to 74.64% and 67.30%, the relative abundance of *Alphaproteobacteria* increasing from 4.29% in the control to 7.95% and 10.62%, and the relative abundance of *Gammaproteobacteria* increasing from 5.63% to 15.10% and 20.07%, respectively. It was suggested that *Alphaproteobacteria* can promote the tolerance of graminaceous plants to elements, such as Co, Cr, and Ni in alkaline soils [34]. Furthermore, some bacteria in *Gammaproteobacteria* can use sulfur as the electron donor to fix CO_2_. The significant increase in the relative abundance of these three groups of bacteria indicated that the application of liquid mulch can promote crop growth and can facilitate carbon cycling in the soil.

As illustrated in Figure 5c, the most significant increase in the relative abundance is *Arthrobacter* of the class *Actinomycetes* after the application of liquid mulch, whose relative abundance increased from 2.78% in the control to 49.89% and 45.82% at 7 days and 14 days of mulch degradation, respectively. Studies have shown that *Arthrobacter* can degrade pesticides and heavy metal cadmium in the soil and promote plant rooting. In addition, the relative abundance of *Nocardioides*, *Pseudarthrobacter*, and *Pseudoxanthomonas* increased. Among them, *Pseudoxanthomonas* has the function of degrading cellulose [35]. *Pseudarthrobacter* can hydrolyze cellulose and is resistant to cold environments and solar radiation. The increased abundance of the above functional microorganisms promoted crop growth through improving soil carbon sequestration and toxicity reduction.

#### 3.3.3. Community Succession of Soil Fungi

**Figure 6:**
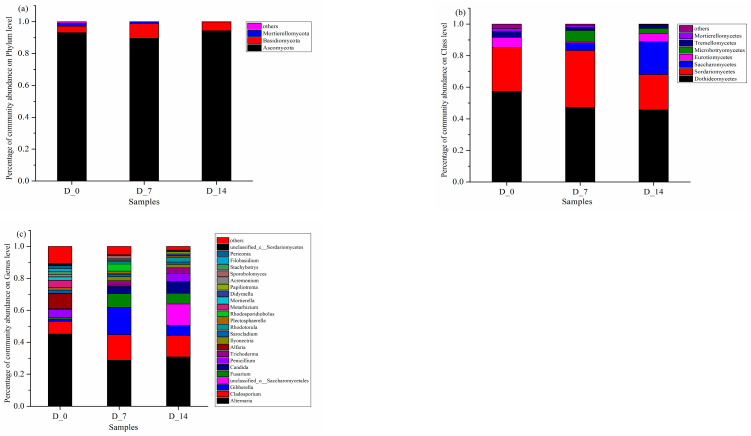
Community composition of fungi at phylum level (**a**), class level (**b**), and genus level (**c**).

As shown in Figure 6a, the dominant fungi in the soil at different degradation times of liquid mulch were mainly *Ascomycota*, *Basidiomycota*, and *Mortierellomycota*. The relative abundance of *Ascomycota*, a saprophytic phylum that degrades wood, food, cloth, and leather in the soil and decomposes plant and animal residues [36], decreased by 3.81% and increased by 1.15% at 7 days and 14 days of mulch degradation compared to the control. The relative abundance of *Basidiomycota* first increased and then decreased with the degradation of the mulch film; it increased by 5.15% and 1.49% at 7 days and 14 days, respectively, compared with the control group. *Basidiomycota* helps plants obtain nutrients from the soil and plants produce glucose through photosynthesis to feed *Basidiomycota* in turn, which can also form symbiotic relationships with insects. The phylum *Basidiomycota* was found to exhibit significant in vitro degradation of hydrocarbons, such as polycyclic aromatic hydrocarbons, persistent organic pollutants, halogenated hydrocarbons, and aromatic hydrocarbons, phenols, explosives, and dyes [37].

As presented in Figure 6b, the dominant soil fungal communities included *Dothideomycetes*, *Sordariomycetes*, *Saccharomycetes*, *Eurotiomucetes*, *Microbotryomyomycetes*, and *Microbotryomyomycetes*, etc., at the phylum level. At 7 days and 14 days of mulch film degradation, the relative abundance of *Dothideomycetes* decreased by 10.05% and 11.66% compared with the control group, respectively. Most *Dothideomycetes* were plant pathogens, causing huge losses to cash crops [38]. The decrease in their relative abundance indicated that liquid mulching could help to control fungal diseases. *Sordariomycetes* are saprophytes that promote nutrient cycling in soil [39]. With the degradation of the mulch film, the relative abundance of *Sordariomycetes* first increased and then decreased by 28.72% and 19.82%, indicating that the fungus participated in the degradation process of the mulch film, and its abundance decreased with the degradation of the mulch film. The relative abundance of *Saccharomycetes* of Ascomycota increased gradually with the degradation of the plastic film and increased by 4.88% and 20.70% at 7 days and 14 days, respectively. *Saccharomycetes* can promote plant growth and reduce the arsenic content in the soil. Thus, it can be concluded that the degradation of the plastic film was beneficial in reducing the content of heavy metals and other harmful substances in soil and promoted crop growth.

As illustrated in Figure 6c, the dominant soil fungal communities were *Alternaria*, *Cladosporium*, and *Gibberella* at the genus level. The relative abundance of *Alternaria* of the ascomycetes was reduced by 16.41% and 14.29% at 7 days and 14 days of mulch degradation, respectively, compared to the control group. *Alternaria* are planted pathogenic bacteria that cause early blight and brown spot disease, which are particularly common in crops in arid regions [40], and the decrease in their relative abundance suggests that liquid mulch is effective in controlling crop diseases. The relative abundance of *Cladosporium*, a genus of ascomycetes, increased by 8.12% and 5.48% at 7 days and 14 days, respectively. *Cladosporium* is mainly distributed in soils with low salinity, and its relative abundance is positively correlated with the content of total nitrogen in the soil [41]. The application of liquid mulch may improve the saline soils, increase the content of nitrogen in the soil, and optimize soil quality. It was apparent that soil nitrogen fixation and disease resistance increased significantly with the degradation of the mulch. The abundance of *Saccharomyces*, *Candida*, and *Penicillium* in the class *Saccharomycetales* increased gradually with the degradation of the mulch film.

### 3.4. Biodegradation Mechanism of Corn-Starch–Sodium-Alginate-Based Liquid Mulch

The soil microbial community succession pattern during the degradation of corn-starch–sodium-alginate-based liquid mulch shows that fungi are the main microorganisms involved in the biodegradation of liquid mulch, and the bacterial community structure and function are optimized during the mulch degradation process. During mulch degradation, the diversity and abundance of bacterial communities in the soil gradually decreased and the abundance of dominant bacteria increased significantly. *Arthrobacter* of the class *Actinomycetes* was the dominant microorganism with the largest percentage of abundance, and its abundance increased by 16.48 times at 14 days of mulch degradation compared with that before degradation. The phylum *Acidobacter* (14 days) decreased by 99.33% compared with that before degradation. The abundance of fungal communities in the soil increased significantly with the degradation of the mulch, among which the *Alternaria* and *Cladosporium* of the *Ascomycete* phylum *Ascomycota* were the most active at the early stage of degradation (7 days). The relative abundance of *Alternaria* of the *Ascomycete* phylum decreased by 16.41% (7 d), and *Cladosporium* increased by 8.12% (7 days) compared with that before degradation. Nakazawa et al. [42] revealed that soft-rotting fungi of the *Cysticercus* phylum can secrete laccase as an oxidation key enzyme, while brown-rotting tannins are capable of chemical reactions without enzymatic involvement. Zheng et al. [43] found that the combined multivariate soil environment strongly influenced the composition and diversity of microbial communities, from which it can be inferred that changes in soil environment may be related to mulch degradation and metabolites produced by microbial growth and metabolic processes. Thus, the specific degradation mechanism needs to be investigated more thoroughly for degradation intermediates.

Related studies have shown that microorganisms of the phylum *Aspergillus* and *Actinomycetes* are important species involved in the degradation of some organic matter and polymeric materials [44,45], which is largely consistent with the dominant phylum species obtained from the above-mentioned community succession of soil bacteria. Huang et al. [46] stated that bacteria are mainly classified as autotrophic and heterotrophic, and there is a complex, non-specific network of interactions between bacterial functional groups.

## 4. Conclusions

The optimal process conditions for the preparation of corn-starch–sodium-alginate-based liquid mulch film involved adding 33.33% sodium alginate, 50% glycerol, and 4% citric acid to corn starch after moist heat modification. FTIR and XRD characterization showed that -OH and -COOH interacted with each other through hydrogen bonding to make the film structure denser, and the tensile strength of the film was 0.145 MPa, and elongation at break was 16.05%. The film was a non-crystalline material with good mechanical properties.

The corn-starch–sodium-alginate-based liquid mulch was completely degraded macroscopically at 25 days. Microbial kinetic analysis showed that the abundance and diversity of bacterial communities decreased and the abundance of fungal communities increased, and the application of liquid mulch favored the dominant microorganisms of *Actinobacteria*, *Proteobacteria*, *Ascomycota*, and *Basidiomycota*. The growth of oligotrophic *Acidobacteria* and some plant pathogens was inhibited. Hence, fungi were the main microorganisms involved in the biodegradation of corn-starch–sodium-alginate-based liquid mulch, and the application of liquid mulch was conducive to the optimization of bacterial community structure and function. The mulch degradation increased the soil organic matter content and the abundance of beneficial soil microorganisms and improved the soil disease and toxicity resistance.

## Figures and Tables

**Table 1:** Experimental design for the preparation of corn-starch–sodium-alginate-based liquid mulch.

Factors	Level				
Modified starch with sodium alginate/Quality ratio	1:1	2:1	3:1	4:1	5:1
Glycerol with sodium alginate/Quality ratio	0.1	0.3	0.5	0.7	0.9
Amount of citric acid added/%	1	2	3	4	5

**Table 2:** EDS element distribution of liquid mulch film.

Element	Mass Ration (%)	Atomic Ratio (%)
C	58.09	64.94
O	41.43	34.78
Na	0.48	0.28
Total	100	100

**Table 3:** Changes in the abundance and diversity of bacteria and fungi during the degradation of liquid mulch.

Microbial Species	Degradation Time/d	Valid Sequences	Shannon	Simpson	Ace	Chao	Coverage
Bacteria	0	48,730	5.43	0.01	1050.51	1048.71	1.000
	7	46,208	2.56	0.26	551.02	522.07	0.997
	14	51,305	2.59	0.23	536.63	441.73	0.997
Fungi	0	39,446	2.64	0.22	130.17	130.00	1.000
	7	45,023	2.57	0.15	149.41	150.63	1.000
	14	71,212	2.56	0.14	172.83	168.77	1.000

## Data Availability

Not applicable.
